# Letter to the Editor: I Spy With my Little Clinician's Eye (CE) …

**DOI:** 10.5435/JAAOSGlobal-D-20-00140

**Published:** 2020-11-10

**Authors:** Dinko Nizić

**Affiliations:** Zagreb, Croatia

*To the Editor*: I have read with pleasure an article entitled “Is the clinician's eye a valid and reproducible tool for diagnosing patella alta on a lateral knee radiography?” by Dr. Vaisman et al.^1^ from Chile, published this month in your journal. I hereby express my admiration for a wonderful piece of science they have produced. However, because there was a mention of our article,^2^ I would like to take this opportunity to inform the authors and the readers of some interesting points raised.

The authors stipulated that we did not report sensitivity and specificity of the new reference line. This is true. We treated all methods as equal, and none as the benchmark. In fact, to this day, no benchmark exists in diagnosing patella alta, as the authors themselves assert in the Introduction section. If we had had a benchmark, we would have reported the indices of diagnostic validity. Although the study was conducted nearly eight years ago, I still have the original data. Based on these data, I have constructed a single table (table 1) consisting of two 2 × 2 frequency tables comparing the binary scores of the new reference line with that of the Insall-Salvati (IS) and Caton-Deschamps (CD) ratio. Because for each knee there were four binary scores by two raters, if three of four were positive, the score was considered positive and if three of four were negative, the score was considered negative (no ties). To begin with, no statistically significant difference was observed between the marginal frequencies (Cochran Q test: n = 64, Q = 4.3, df = 2, *P* = 0.115). If we had considered IS to be the truth, the sensitivity of the new line would have been 89% (52 to 100) and specificity 93% (82 to 98). On the other hand, had we treated CD ratio as the truth, like it seems that the authors did, the sensitivity of the new line would have been 100% (63 to 100) and specificity, again, 93% (83 to 98). Positive predictive value (PPV) would have been 67% (35 to 90) with both IS and CD, whereas negative predictive value (NPV) would have been 98% (90 to 100) with IS and 100% (93 to 100) with CD. Therefore, both times our indices of diagnostic validity would have scored higher than Vaisman et al. reported as averages for their clinician's eye (CE) (77% sensitivity and 92% specificity). I sincerely thank the authors for making this remark, else I would not have felt compelled to make this calculation public since, as I wrote, back then we did not feel there was a method reliable enough to represent the truth. We were modest in thinking that the new reference line was equally good or bad as the other four methods regarding accuracy, although very simple and reproducible.

**Table 1:** 2 × 2 Frequency Tables Comparing Binary Scores of the New Reference Line in Diagnosing Patella Alta With That of the Insall-Salvati and Caton-Deschamps Ratios

	IS			CD		
	+	−	mf	+	−	mf
New reference line						
+	8	4	12	8	4	12
−	1	51	52	0	52	52
mf	9	55	64	8	56	64

CD = Caton-Deschamps ratio, IS = Insall-Salvati ratio, mf = marginal frequencies

It is likewise unknown whether less-trained observers might have achieved a less favorable result with using the new reference line, and we hinted this in the study. However, all one needs to construct a line is to know very basic school geometry such as lines and ellipses; at least, it is so in Croatia. Thus, I would feel it seems reasonable to assume it would be rather unlikely a less-trained professional, such as, say, a resident, would have any particular issues embarking on this simple geometric task.

Moreover, in Methods section, under Study design, the authors have stated that the CE method “(…) consists of a subjective estimation of patellar height to confirm or rule out PA (patella alta).” I am a bit puzzled here: what did the authors specifically look at or look for while looking at the images? Did each of them evaluate each of the known ratios by a naked eye, without calculation, and then these evaluations were compared with CD ratio as a benchmark? If not, what is the exact content of the CE method? *Experience* is a broad term and could mean different things to different people, even on an equal level of expertise. The same goes for the word *intuitive*. Among other things, our vision is a measuring instrument, and with standardization, others might see what you saw. Otherwise, replication becomes difficult.

Although we had a greater sample size (64 compared with 46), wider age range (16 to 85 years compared with 20 to 40 years), and the range of knee flexion (1.6° to 79.1° compared with 7.6° to 70°) proving the line could be stable in a more variable age group and imaging conditions, I would be hesitant to call this line a benchmark anytime soon. It might be just as easily overrun by a better method in the future. However, until that time, here is an interesting thought: in an independent way, in two different parts of the world, both Dr. Vaisman with his team in South America and Dr. Pervan and I earlier in Europe seemed to substantiate the fact that vision alone has remarkably high specificity in diagnosing patella alta. By the same standards, the new reference line would seem to have the same specificity, yet higher sensitivity (at least 89% compared with CEs 77%). Of all the methods currently at disposal to diagnose patella alta, the line does not need a calculation, but it might not even need to be drawn on the radiographic image—it could be drawn in the eye of the beholder, the CE. Could this mental projection of the new reference line on the lateral knee radiographic image improve the sensitivity of the CE in diagnosing patella alta while retaining a high level of specificity?

I congratulate to Dr. Vaisman and his team and encourage them to keep up with their work.
